# Community Pharmacists’ Perceptions of Patient Care Services within an Enhanced Service Network

**DOI:** 10.3390/pharmacy8030172

**Published:** 2020-09-16

**Authors:** Christopher J. Daly, Bryan Quinn, Anna Mak, David M. Jacobs

**Affiliations:** Department of Pharmacy Practice, University at Buffalo School of Pharmacy and Pharmaceutical Sciences, Buffalo, NY 14214, USA; bryanqui@buffalo.edu (B.Q.); amak2@buffalo.edu (A.M.); dmjacobs@buffalo.edu (D.M.J.)

**Keywords:** community pharmacy services, medication therapy management, sustainable business models for community pharmacy services, social determinants of health, community pharmacy enhanced services network

## Abstract

Background: Pharmacists are positioned as an accessible source of patient care services (PCS). Despite the adversity community pharmacies continue to face, the expanding opportunity of offering PCS continues to be a pathway forward. Objective: To identify community pharmacists’ perceptions to deliver PCS within an enhanced service network. Methods: One-on-one semi-structured phone interviews were conducted as part of a mixed-methods approach. Interview transcripts were analyzed using a consensus codebook to draft thematic findings. Participants were recruited from an electronic survey targeting community pharmacists from the New York chapters of the Community Pharmacy Enhanced Services Network (CPESN). Results: Twelve pharmacists were interviewed with four main themes identified. The majority of study participants were pharmacy owners (92%) devoting an average of 15 h/week to PCS and 8 h/week addressing social barriers. The main themes identified include: (1) perceptions of pharmacy profession, (2) reimbursement models and sustainability of PCS, (3) provision of patient care services, and (4) how PCS address social determinants of health. Conclusions: Offering PCS opportunities for patients is a direction many community pharmacists have embraced and are working to succeed. Ongoing research is needed focusing on community pharmacists’ self-perceptions of the clinical impact and role they hold in an evolving healthcare system.

## 1. Introduction

The rise of chronic disease necessitates increased access for patient care services (PCS) while controlling the cost of care, and ultimately improving healthcare outcomes [1]. PCS are utilized by healthcare providers to promote a focused approach on a patient’s health in regard to a sense of control and efficacy to all contributing factors of their health [2]. PCS in the context of community pharmacy are those services performed that are not product or dispensing related. These services are focused on improving patient-specific clinical goals [3]. This patient-centric approach strategizes to improve the relationships patients have with their providers while concurrently addressing health concerns by diagnosis, as well as concerns beyond the clinical constructs such as social determinants of health. This type of approach in the American healthcare system is met with a high percentage chance of conflict, compared to other Organization for Economic Co-operation and Development (OECD) countries, of patient medical coverage, higher medical costs, and access to healthcare [4]. To address the opportunity, there has been a continuous shift in how community pharmacies deliver services to the public. This shift is emphasized by horizontal integration of pharmacies into larger healthcare entities or vertical integration of new care services within the pharmacies [5]. The role of a community pharmacy has become pertinent to the public, especially to those with multiple chronic conditions and complex medication regimens [6]. The pharmacy is one of the most accessible and frequently used front-line healthcare entities. Fay et al. highlighted, when analyzing North Carolina Medicaid claims data, that the portion of the population most in need of medication management visit their local pharmacy 20–35 times annually [7]. These high-risk patients were targeted for coordination efforts by pharmacists having the ability to complete multiple patient interactions throughout a work day [8]. Current improvements that pharmacies are adopting to include training on the social determinants of health to aid diverse communities, collaboration with local health and social services, and being proactive with current incidents such as COVID-19 and how clinics can actively combat them [9]. Formulating newly defined roles for pharmacists may help address some of the issues that patients face, especially increasing access to services covering a wider spectrum of health-related issues aside from a clinical diagnosis.

The role of pharmacists has been viewed in a multitude of ways, both clinically and to the public. American pharmacists are attempting to change the view of which the community believes that they are only there to provide medication. The on-going shift being mentioned is one from a “product-centric” model to a “patient-centric” model [10]. With increased training in areas such as the social determinants of health, providing care that extends beyond the spectrum of the clinic doors is a small portion of what community pharmacies are implementing into their workflow [5]. Examples of these types of services are referrals to health and social services, patient education on medications and conditions, or care programs where patients can rely on staff care managers from the pharmacy [10]. Creating a safer environment for patients to seek clinical attention during important times, especially with regard to the complex medical world, could help change how patients view their visits to community pharmacies.

The on-going shift to patient-centric and service-based models continues to be explored by American community pharmacies. There are still many barriers that could prevent this from being accomplished. The most prominent barrier would be community pharmacies not receiving reimbursement for PCS through third-party payers since they are not currently seen as providers per the Medicare Part B program [11,12]. Currently, both private and public providers of insurance do not regularly pay for PCS [13].

Contracts for prescription payment are facilitated by healthcare intermediaries referred to as Pharmacy Benefit Managers (PBMs). The main source of reimbursement for American community pharmacies involves two components: dispensing fee and prescription drug cost [14]. Almost all private and public (e.g., Medicare Part D) third-party payers through PBMs negotiate reimbursement rates with the community pharmacy. Community pharmacies lack leverage when looking to establish fair and equitable rates that cover both the drug costs and dispensing fee components of pharmacy reimbursement [14]. This peril has left individual community pharmacies little choice when accepting low rate contracts to maintain patients while not violating antitrust laws. The impact of PBMs lack of transparency and inadequate regulation is critical to the financial viability of future community pharmacies [15]. The National Community Pharmacists Association has called for the modernization of pharmacy reimbursement to protect patient access to the state Medicaid programs [16]. A cost of dispensing report was commissioned showing pharmacies are currently inadequately reimbursed. The primary argument of the report outlined Federal Medicaid rules require state Medicaid fee-for-service programs to pay pharmacies dispensing fees that cover pharmacies’ costs of dispensing prescription medications and providing related benefit and coverage services [16,17]. It was stated that dispensing fees are vital to account for community pharmacies’ overhead costs to do business [17]. It is important to note that dispensing fees are separate from care coordination activities that are considered PCS.

Other underlying barriers are that some pharmacies currently lack the proper layout to accommodate certain PCS, such as a separate and confidential room for patient-specific medical consultations [11]. There is also the concern over the lack of proper resources and available technology to have productive services such as access to electronic health records and the ability to document these patient services [7].

Despite the added stressors and adversity community pharmacies face, the push to change the dynamic of workflow and how the community views what a pharmacy is has taken precedent. Pharmacies have the ability to advocate the beneficial use of PCS, to professional organizations for steps towards legislative change. This study has worked to exhibit where current barriers exist and assess the readiness and commitment levels by utilizing a one-on-one interview approach with pharmacists. Several qualitative studies have used this method rather than the traditional quantitative survey approach due to its ability to draw out themes through sentence context. Therefore, the objective of this study was to identify community pharmacists’ perceptions to deliver PCS within an enhanced service network.

## 2. Materials and Methods

### 2.1. Study Design

A qualitative study was conducted using one-on-one interviews as part of a mixed-methods approach. Participants originally completed a 35-item cross-sectional, electronic survey designed to evaluate the level of commitment and readiness among independent, community pharmacies to deliver PCS and address patient-level social barriers. Inclusion criteria included if they were a full-time licensed pharmacist employed at a non-chain, independent community pharmacy, if they were directly involved in PCS delivery or involved in the process, or if they were knowledgeable on and help set up PCS at their community pharmacy. To recruit participants, the survey was distributed to the New York chapters of the Community Pharmacy Enhanced Services Network (CPESN NY). A copy of this survey can be accessed as an online Supplement.

A total of 48 potential interviewees self-identified based on their willingness to provide a follow-up interview from the initial survey. Potential interviewees were engaged to participate via telephone by members of the research team (BQ and AM). Participants were chosen based on geographical location to provide a diverse pool of interviewees. Representation consisted of all CPESN NY chapters: New York City, Upstate New York, and Western New York. The authors aimed to recruit participants from the three chapters and stopped recruitment after consistent findings and perceptions were reached.

CPESN NY was launched in 2017 and consisted of three separate original chapters (New York City, Upstate New York, and Western New York). The network is part of a nationwide organization of pharmacy networks structured to advance community-based pharmacy practice. They empower community-based pharmacies that are deeply rooted within their community by fostering their ability to provide high-quality, patient-centered enhanced services [3]. Enhanced services are described as services that transcend conventional requirements of an outpatient pharmacy program contract that are focused on improving clinical and global patient outcomes [3]. Examples include: home delivery with patient status review, medication synchronization with clinical review, and adherence packaging with patient coaching. The prime objective of these clinically integrated networks is to be able to negotiate as a larger entity for payment of PCS. Based on the commitment to this mission, this group was a favorable target to focus this assessment.

### 2.2. Data Collection

Two trained research assistants (BQ and AM) conduced the interviews, six each. The interviews were conducted via phone to facilitate participation across the state. The personal interview format was chosen to best understand individual perspectives about the current state of community pharmacy practice and opposed to consensus statements. The interviews were executed based on a semi-structured interview guide (Appendix A) as this allowed for flexibility throughout the interview and for pharmacists to expand on their responses. The interview guide was developed by the research team after seeking expert input and completing a literature search.

At the beginning of the interviews, the goals and objectives of the project were shared with the participant along with a statement declaring that the call would be confidential and recorded with the participant’s consent. All of the participants who were interviewed agreed to a confidential phone interview that would be recorded and transcribed. All interviews were conducted in English, digitally audio recorded, and transcribed verbatim. Either BQ or AM were present to conduct the interview and record the conversation. Confidentiality was protected by de-identifying the data after collection and protected on password-protected research computers. This study was approved by the University at Buffalo Institutional Review Board (IRB #00003033).

All interview data was collected from February 2018 to March 2018 using the semi-structured interview guide. The main questions were designed to support the study objectives regarding community pharmacists’ commitment to delivering PCS. Follow-up probe questions were designed to capture the pharmacist’s unique insight on PCS and how services related to the patient population. Examples of these questions include: “What patient care services are you providing in addition to dispensing, and how are you being reimbursed?”, “What barriers are preventing you from providing patient care services?”, “What services would you incorporate into your pharmacy that would allow you to better serve underserved populations”, and “What barriers are preventing you from providing adequate care to underserved populations at your pharmacy?” The complete interview guide is provided in Appendix A.

### 2.3. Data Analysis

During the process of data analysis, the researchers most closely involved in data collection and the early stages of analysis (BQ and AM) met with senior members of the research team with extensive content (CD) and methodology (DJ) experience. Common categories broken down into themes and sub-themes were presented for review. Further interpretation of key details led to new pathways of inquiry. The collective insight of the data collectors to members of the full team with a wider perspective of methodological and content was formed. The following details the process taken.

At a mid-point of the analysis of the qualitative data, a research meeting was conducted of all team members examining five de-identified transcripts. Codes were created by the members of the team most closely involved in data collection and analysis (BQ and AM). As an independent check on the assignment of codes, five de-identified transcripts were reviewed by other team members (CD and DJ). A set of codes were generated based on the interview guide. Two of the authors (BQ and AM) read through the data files and independently coded each of the five original interview transcripts. Each coder developed preliminary ideas about themes [18]. After coding these files independently, the authors met to review and discuss points identified in coding and developed a codebook to be used for the remaining interviews. The two authors (BQ and AM) coded the remaining de-identified interview data collectively with senior member team review. After an additional seven interviews were conducted, transcribed, and coded, the research team met to discuss consensus themes [19]. The research team agreed due to the similar themes using the same codebook, that data saturation was reached. No additional interviews were sought. After the transcripts were coded, a summary of findings was sent to three of the interview participants to provide feedback. The aim of this process was to make sure the interpretation of the findings was consistent with current experiences. The interview participants agreed and did not provide any changes to the findings. The authors followed the consolidated criteria for reporting qualitative studies (COREQ) guidelines for reporting qualitative research [20]. Further details can be found in Appendix B.

## 3. Results

### 3.1. Study Participants

A total of 12 community pharmacists practising in independently owned community pharmacies involved in PCS participated in the study. Interview times ranged from 27 min to 99 min with an average length of 45 min. The majority (11) of the study participants were pharmacy owners as shown in Table 1. It is important to note that the non-owner pharmacist participant did not have differing views from the owner pharmacist participants. Responses were collected from the three geographically diverse chapters: New York City (2), Upstate New York (6), and Western New York (4). Little differences in views of pharmacists were identified based on geographical location. Therefore, to avoid the risk of a potential confidentiality breach, pseudonymised codes followed the current format (e.g., Pharmacist 1, Pharmacist 2, etc.).

Over half of the community pharmacies (*n* = 7) had a weekly prescription count of >1200. The average time devoted to PCS was 15 h/week and pharmacies on average spent 8 h/week addressing social barriers. Respondents noted a high percentage of pharmacy patients experiencing social-related barriers with high medication costs and low income being the most common identified barriers. Both pharmacist and entity characteristics were collected and outlined and are summarized in Table 1.

### 3.2. Perceptions of the Pharmacy Profession

After thorough analysis of the data, four themes were presented to the research team: (1) perceptions of pharmacy profession, (2) reimbursement models, (3) provision of patient care services, (4) social determinants of health. These were further broken down into subthemes and explored further. Table 2 provides a summary description of the subthemes and are explored in greater detail. Appendix C is a supplemental table looking at further details of selected quotes highlighting subthemes identified.

Patients have a varying degree of outlook on how a pharmacist’s role may impact their overall healthcare. Patients perceive pharmacists to be trustworthy and knowledgeable. PCS such as extensive patient counseling, blister packaging, immunization, and medication therapy management are improving the perception of the profession. However, most patients are still unaware of the pharmacist’s role beyond dispensing and into patient care:

“I don’t believe most people know what we do. Most think that we are still just in charge of medication.” [Pharmacist 2]

Among healthcare providers, the perception of pharmacists is improving with increased opportunities for pharmacist involvement through PCS. However, resistance and pushback remain from some providers towards collaboration as pharmacists need to continue to prove value.

“We mostly just see resistance. It is very hard for us to have a relationship with the providers in the area. We are trying but it does not seem like they want to collaborate. They always ignore our calls and do not call back about our recommendations and/or questions.” [Pharmacist 12]

Pharmacists must promote PCS to raise awareness of the profession’s abilities. This is facilitated through advocating for the profession. Both the public and providers need to leverage campaigns to be educated on the value that a pharmacist can provide by implementing PCS. Pharmacists also promote PCS to raise awareness of the profession’s abilities. Pharmacists also encourage a proactive approach in expanding scope by advocating for legislative change:

“…We try to talk to our senators, and people that pharmacists can do more and are an important part of the medical circle. Educating decision makers and law makers that we should be used more and reimbursed too. In the end to keep people out of the hospital and lower medical spending for insurance companies and our state-run plans.” [Pharmacist 7]

Public perception of pharmacists is important. One of the best ways to make patients aware of the pharmacist’s capabilities is by developing a personalized relationship with them. This can be done via counseling and other regular outreach:

“We believe that service above all is what patients want to experience. They want a good experience from when they walk up to the counter to the minute they walk out the door. They want a friendly helpful and informative experience. What we do is try to be as comprehensive as possible by offering whatever information the patient may want and letting them know that we are available 7 days a week for them. There is always a pharmacist available to speak with the patient and there is never a wait to speak with the pharmacist. We also offer a HIPAA compliant texting program right to the pharmacist’s screen so you can chat with the pharmacist.” [Pharmacist 11]

### 3.3. Reimbursement Models

Participants stated that dispensing medications as a sole source of income is unsustainable due to declining reimbursement rates. Community pharmacies are not adequately reimbursed if at all, for the provision of PCS. This is especially the case with patients presenting with social determinants of health barriers. Costs to provide PCS and declining reimbursement decreases the ability to provide new PCS. There is a need for sustainable sources of income for providing PCS:

“…reimbursement, there’s only so much you can do for free. Pharmacists aren’t cheap, and our time is very scarce. …it’s disappointing when they don’t get paid for their time.” [Pharmacist 7]

Moreover, pharmacies are not adequately reimbursed from pharmacy benefit managers (PBMs) for the provision of PCS. The increased cost of providing PCS and the decreased reimbursement for medication dispensing hampers the ability to provide PCS:

“The hardest thing is implementing something that you don’t see a return on as far as profitability. With the way things are now in pharmacy, these PBMs are squeezing us tighter and tighter. It seems like every month there’s another PBM cutting reimbursement rates and it makes staffing difficult if there’s not some sort of model in place to be reimbursed for these services.” [Pharmacist 8]

With the goal of increasing their profits, PBMs are getting in the way of the pharmacist–patient relationship. Low imbursement rates for medication dispensing and PCS coupled with deleterious requirements, such as mail order delivery, prevents appropriate patient care follow-up by the pharmacist. This contributes to poor health outcomes:

“From a provider point of view, a lot of that has to do with PBMs and insurance companies. On one hand, we try to do things like MTM that are very beneficial to patients, but on the other hand they [PBMs/insurance companies] force their members to go to mail order and [go into] other non-personal things that cheapen and diminish the value of a pharmacist. They’re [PBMs/insurance companies] basically telling their patients/members: we’re just going to have your medications delivered to your door without any follow-up care.” [Pharmacist 8]

One of the potential sources of income for providing start-up support for PCS is grant money. Organizations such as Community Pharmacy Foundation (CPF) fund grants for ideas that advance the practice of pharmacy in the community setting [21]. Grants such as those provided by CPF can become a foundation for PCS pilot projects. The pilot projects can serve as a proof of concept in order to validate the PCS and augment them into the community setting on a broad scale via entities that are responsible for the patients’ health related cost:

“I’ve heard of pharmacies receiving grants, which would be a huge benefit. We were in a blood pressure one which provided a fair amount of money for pharmacist’s assistance. Any government-funded programs or initiative where there is proper reimbursement for a pharmacist’s time would be some resources that we would be interested in.” [Pharmacist 9]

Funding for PCS can also be secured by contracting with managed care organizations:

“We are in the process of contracting with a managed care organization, where the insurance company will be paying us $20 a month for each of their patients for the [adherence] blister packaging.” [Pharmacist 5]

The focus is shifted to plan sponsors who ultimately pay for the healthcare services (e.g., employers, government and individual consumers buying their own health insurance). Future models rely on securing contracts through pharmacy organizations such as Community Pharmacy Enhanced Service Network (CPESN) [22]:

“…I have also become an early adopter of the Community Pharmacy Enhanced Services Network…CPESN cannot help me with the PBMs, rather it helps me go around the PBMs. CPESN is a clinically integrated network of networks. The clinically integrated network status gives me the legal ability to negotiate contracts with payers for my network.” [Pharmacist 11]

A network must have enough pharmacies in it to adequately cover the patients of a plan sponsor. Therefore, ideal future models would also need network adequacy to secure fair contracts for the pharmacies. To this end, active participation from pharmacies and pharmacists would be required:

“We really need to have everyone doing CPESN networks to help enhance pharmacy networks to provide better quality care.” [Pharmacist 10]

### 3.4. Provision of Patient Care Services

Participants outlined how inadequate resources to devote to Patient Care Services (PCS) will present barriers. These include: the high cost of providing, inadequate reimbursement for PCS, and lack of time. Patients face poor health literacy, especially in underserved areas where PCS are needed the most. Currently, there is a lack of integration of pharmacists providing PCS into the healthcare system. Pharmacists also have limited access to a patient’s health record through state-run data sharing collaboratives such as lab results. This assists in the provision of PCS and how a pharmacist can impact care. Appendix C provides additional perceptions on the provision of PCS. This highlights the process required to access patient clinical information:

“A third one would be having more tools and accessibility to information out there, and being integrated into healthcare system. I just enrolled in HIXNY [This is a regional health information exchange platform] New York where I have access to patients’ notes when they get admitted/discharged at a hospital. It’s a great tool that’s more centered around the Albany area, so not all the hospitals participate and feed the data into the system. HIXNY is health information portal where the patient signs release form to allow access to their HIXNY information. It allows access to discharge notes, lab values so it’s easier to do med recs and to bill for med devices.” [Pharmacist 4]

Narrowing insurance formularies along with increased prior authorizations for medications limit patient access to medications. Increasing mandatory prescription mail order prevents appropriate face-to-face follow-up care and contributes to medication adherence issues. This adversely affects patient health outcomes.

“…insurance approved therapy, but the provider requested he fill through my pharmacy. The insurance had a limited formulary and wanted patient to go to a specialty pharmacy for treatment…” [Pharmacist 8]

There are various elements that need to be incorporated when integrating PCS into a pharmacy. PCS can either be integrated straight into the daily operations, or be removed completely out of the standard workflow and treated as a separate entity. However, it should be noted that in some cases PCS such as med sync program can make the filling and staffing process more efficient:

“Our med sync program has dramatically changed our work flow. Rather than our pharmacy being reactive and not being in control of our workflow, we are very proactive with our work load. For our med sync, we only fill prescriptions for med sync patients twice a week, so we are able to appropriately staff those days. We have lighter staff at other points in time which helps with work-life balance. It also lets our staff know that they will have adequate back-up on busier days. They also know that this isn’t anything that needs to be dealt with in the next 10 min because the patient won’t be here until another 8 days. It relieves a lot of stress in that we are very proactive with our workflow.” [Pharmacist 6]

Technology is a driving force behind the efficiency and effectiveness of PCS. Platforms and dispensing software systems are becoming increasing more capable of executing PCS workflow:

“First step was to gather information from others doing it. We started with small steps and leaned on our pharmacy software in our system… Now we have ability to identify patients who are in greater need of services. We can get a report of patients that would benefit from clinical services. Their system can put in filters to show patients who would best benefit from opioid services and help prevent opioid addiction.” [Pharmacist 3]

PCS require devoted resources, the most important being staff time to delegate to such operations. Staffing will vary between different community pharmacy settings depending on the approach taken. This commitment will depend on proper task delegation (e.g., tasks not requiring pharmacists and given to others) and providing PCS focus to a select number of pharmacy staff.

“Having a 3:1 tech to pharmacist ratio. Similar to how you have NPs, nurses, and other professions to help doctors take care of easier tasks so that doctors can focus on tougher patients. I think we can do that with techs and take some of the pressure off pharmacists, by letting techs handle more of the phone calls and more billing questions.” [Pharmacist 3]

Outcomes from PCS need to be quantified in order to be appropriately compensated by plan sponsors looking for positive health outcomes. PCS such as adherence packaging, medication synchronization, and medication therapy management can lead to increased medication adherence. Enhanced patient counseling and wellness programs can lead to increased patient education. Successful implementation of PCS will lead to positive outcomes:

“We’re trying to gather some data right now on people’s HgA1c (glycated hemoglobin) values. We do have weight loss clinic next door to the pharmacy that we partner with. We do have conversations with people that come in who are diabetic or prediabetic that comes in with their first metformin script. Or we see patients with metabolic syndrome (hypertensive, high cholesterol, diabetic) and we have a conversation at the counter. Our program is still in the beginning phases, but we have 10 people where we made substantial improvements in their HgA1c values, cholesterol, and blood pressure. I think overall, that’s where we’re making a big impact right now that we can actually measure. We have other things that we can’t really measure. We’re trying to quantify their improvements. For example, losing 10% of their body weight: how much does that improve their numbers?” [Pharmacist 4]

### 3.5. Social Determinants of Health

Participants felt that developing a personalized relationship with patients is important in order to improve patient outcomes. Personalized relationships allow pharmacists to determine the appropriate PCS to align based on patients’ barriers to healthcare:

“I think we personalize care more. When we go out and talk with patients, it’s not quick. We get more into a conversation with the patient. Most of it is more of a personalization type of care. We ask more questions like what got you here, how do you feel to get more information out of them. I think they appreciate that, when they leave they feel a little more confident.” [Pharmacist 5]

Identifying barriers to patient’s social determinants of health is essential in providing appropriate PCS. Late/early refills or medication stockpiling may indicate low health literacy, where a lack of transportation or limited access to primary care providers, leads to accessibility concerns. Inability to afford the medication co-pays may indicate inadequate insurance coverage or gaps in coverage. Pharmacists can help patients understand their health insurance plans better and work on ways to save money. Other means such as coupon cards, contacting drug manufacturers and finding lower cost therapies can and should be used in order to overcome patient barriers:

“… We are in a low-income area and such a large percent of my population is Medicare, Medicaid or dual eligible. I start every encounter with a patient thinking that there are going to be barriers to their ability to receive healthcare. I think about the services we offer: delivery, compliance (adherence) packaging, CPESN programs and when I have a conversation with a patient I think about what services they might need or would benefit from.” [Pharmacist 1]

## 4. Discussion

The results of this qualitative analysis identified several themes outlining the commitment of community pharmacists to deliver PCS. The themes also highlight the unique challenges that American pharmacy practice faces in the next stages of development. It is looking at these themes that will help to demonstrate the various ways pharmacy practice can move forward. Previous community pharmacy qualitative work describing community pharmacy practice transformation [23], patient care services [23,24,25,26], provider collaboration [13], and alternative payment models [1] shows the current dynamic model evolution. Identifying key areas for community pharmacy practice improvement suggests the potential for the expansion of PCS. Challenges present in the current time outlined by our participants show that there are many obstacles to overcome. Our results suggest community pharmacists, while willing to expand PCS, are met with a host of challenges as next steps. We have identified four overarching themes presented by our subjects: pharmacy profession, reimbursement models, provision of PCS, and social determinants of health.

### 4.1. Perceptions of the Pharmacy Profession

The current perceptions of the pharmacy profession vary depending on the perspectives of patients or providers. Patients are eager to engage pharmacists for personal health concerns due to accessibility, personal relationships and knowledge of medications. Patients still lack a deeper understanding of how pharmacists may provide care through a diverse array of PCS. This is consistent with other study findings. In Smith et al., patients simply do not seek medication counseling information from community pharmacists [13]. The reason for this is the unclear perceptions of pharmacists in non-dispensing roles.

The traditional model of pharmacy dispensing is a directed demand model downstream of providers with prescriptive authority. By instituting PCS, this is increasing pharmacist involvement in the delivery and outcomes of patient care. The gap in understanding of the role the community pharmacist plays on the healthcare team was a prime finding. Establishing provider engagement and collaboration is needed when pharmacists plan to institute PCS. Marketing these services, focusing on provider engagement will drive next steps in this development. Turner et al. emphasize how pre-existing relationships, repeated interactions, and multiple relationships (e.g., collaborating on two mutual clinical interventions) can strengthen provider collaborations with PCS [1]. Our findings were consistent with these as community pharmacists need to develop relationships with providers in order to expand the acceptance and uptake of PCS.

Advocacy and professionalism are charges that many community pharmacists share passionate views about. Due to the regulatory nature of the American pharmacy profession, participants shared a common desire to participate and engage others to become involved [27]. There has been incremental change made but more progress is needed in order to practice at a desired level. The top issues facing the community pharmacy today are PBM reform (e.g., audits, reimbursement methodologies, mandatory mail order) and scope of practice (e.g., provider status, PCS expansion, and collaborative practice opportunities) [28,29].

### 4.2. Perceptions on Reimbursement Models

The development of sustainable reimbursement models for PCS are a focus for many community pharmacies [30,31,32,33,34]. The current PCS models have been labeled as unsustainable for the following reasons: non-adequate reimbursement, no source of revenue for PCS, lack of collaborators (e.g., granting agencies, managed care organizations) to support the provision and payment of PCS [13]. Smith et al. detailed how certain barriers of entry exist, leading to difficulty in starting new collaborations. Limiting factors include: community pharmacist clinical qualifications, provider participation, patient risk stratification, effective and transparent metrics, and a lack of standardized fee structure for pharmacists.

Cost savings seen with PCS do not directly relate with current reimbursement models [35]. The development of these models will be key determining reimbursement options for community pharmacists offering PCS [36]. Doucette et al. took an in-depth look at how an in-house developed continuous medication monitoring (CoMM) program could be implemented, analyzed, and scaled to work in a community pharmacy workflow. CoMM at twelve months, when compared to the standard of care, resulted in a per-member per-month decreased cost of at least $298 and an adherence increase of 2.6%, as measured by a Proportion of Days Covered (PDC) score. PCS integration was noted as a feasible process and able to be implemented around the time of dispensing [33].

Future models are emerging and offering community pharmacies the opportunity to evaluate business strategies [11,37]. One such model is joining a CPESN network whose primary goal is to reduce medical expenditure and improve patients’ health-related outcomes with the provision of PCS [3]. Community pharmacies through these networks demonstrate the ability to impact care and justify reimbursement from entities supporting the medical mission [7,22]. This was identified by our participants as a way to potentially increase contracting opportunities for CPESN pharmacies to increase PCS reimbursement.

### 4.3. Perceptions on the Provision of Patient Care Services

Community pharmacists face many obstacles when delivering PCS. These non-traditional services bring great potential and are valued by patients. They can also form operational concerns when it comes to implementation. Chui et al. reviewed with pharmacists the barriers and facilitators when providing PCS. They found among community pharmacists that implementation requires a multifactorial plan. Training of important skills and competencies is just part of the picture [23]. Community pharmacies, assisted by CPESN initiatives (e.g., Flip the Pharmacy), are provided practice transformation resources that assist in the provision of delivering PCS [38].

Access to quantifiable data supporting health-related outcomes for PCS is both an opportunity and concern facing community pharmacists [39,40]. The medications adherence metric Proportion of Days Covered (PDC) is an indirect measure for reporting health outcomes [41]. Community pharmacists have been shown to positively improve this metric, leading to health improvement [42,43]. However very few contracts are linked to positive revenue surrounding PDC [44]. It is important to recognize the perceived value of delivering PCS: increased medication adherence, clinical effectiveness of medications, accessibility (e.g., immunizations, delivery), and patient education (e.g., counseling, wellness programs) [45].

### 4.4. Perceptions on Social Determinants of Health

Community pharmacists faced barriers to patient care on a daily basis. Being one of the most accessible healthcare providers, community pharmacists are uniquely positioned to connect individuals to resources through relationship development [8]. This key attribute helps promote personalized care management that aims to help patients and families navigate the healthcare system [46]. Patient barriers to care that community pharmacists face on a daily basis range from the following: low health literacy, low socioeconomic status, insurance coverage limitations, lack of transportation, and limited access to primary care. To directly combat these barriers, community pharmacists are positioned to offer customized, unique solutions to help overcome these gaps [7]. They include, but are not limited to: offering PCS, hand-to-hand delivery, medication synchronization, adherence packaging, preventative medicine (e.g., immunizations), and patient education (e.g., smoking cessation) [3].

Medication synchronization can reduce the number of trips a patient needs to make to the pharmacy. It can also cut back on the additional delivery costs if the pharmacy is providing free delivery [47]. Adherence packaging can help patients better understand their therapy and achieve compliance [43]. Enhanced patient counseling can help patients that have trouble understanding their therapy. It can clarify misconceptions about chronic disease management and improve medication adherence due to increased patient health literacy [48,49]. Wellness programs designed for chronic diseases such as diabetes and hypertension can improve the results of medication therapy [50,51,52,53].

### 4.5. Limitations

Pharmacists interviewed were required to have knowledge of internal operations, workflow and implementation of PCS. Due to the complexity of PCS, interviewee bias can play a role in varied perceptions. Participation was based on convenience sampling and it is possible that pharmacist perceptions may vary from geographical and population centers. Eleven of the twelve community pharmacies that were interviewed were pharmacy owners. Other limiting factors include recruitment from a single community pharmacy setting (e.g., independent pharmacies) and state of practice (e.g., New York). Limited global generalisability is identified due to these factors. The interview guide only represents a focused view of issues facing community pharmacies. Unique challenges facing the future progression of the American community pharmacist are shared and expressed through new established pathways (e.g., CPESN NY). The themes identified are shared among pharmacists as common challenges when looking to expand PCS opportunities.

## 5. Conclusions

The opportunities and challenges facing community pharmacists are diverse and complex. As more PCS become available and spread to other community pharmacies, developing and maintaining relationships across a diverse group of stakeholders will be fundamental. Offering PCS opportunities for patients is a direction many community pharmacists have embraced and are working on the plan to succeed. The approach to this pathway through clinically integrated enhanced service networks has been adopted as one of the latest strategies to overcome set obstacles. Ongoing research is needed focusing on community pharmacists’ self-perceptions of the clinical impact and role they hold in an evolving healthcare system.

## Figures and Tables

**Table 1:** Demographics and community pharmacy characteristics of participating pharmacists and their pharmacies.

Demographics (n = 12)	*n*	(%) *
**Pharmacist Role**		
Owner	11	91.7
Supervising Pharmacist	8	66.7
Manager	5	41.7
**Pharmacy Advocacy Organization Membership of Pharmacist**		
Pharmacists Society of the State of New York	12	100
Local Pharmacists Society of the State of New York Affiliate	10	83.3
National Community Pharmacists Association	9	75.0
American Pharmacists Association	3	25.0
**Community Pharmacy Enhanced Services Network of Pharmacy**	12	100.0
CPESN—Upstate New York	6	50.0
CPESN—New York City	2	16.7
CPESN—Western New York	4	33.3
**Community Pharmacy Characteristics (n = 12)**		
**Weekly Prescription Count of Pharmacy**		
<200	0	0.0
201–400	1	8.3
401–800	0	0.0
801–1000	2	16.7
1001–1200	2	16.7
>1200	7	58.3
**Social Barriers Present at Pharmacy reported by Pharmacists**		
High medication costs	10	83.3
Low income	10	83.3
Low education level	9	75
Lack of insurance coverage	9	75
Transportation	7	58.3
Lack of primary care physician	6	50
**Percentage of Patients Experiencing Social Barriers reported by Pharmacists**		
0–10%	2	17
11–30%	5	42
31–50%	2	17
>50%	3	25

* All values are expressed in total number (*n*) and percentages (%), unless indicated otherwise. Abb. CPESN, Community Pharmacy Enhanced Services Network.

**Table 2:** Summary description of interview participant perception themes.

Themes	Subthemes	Summary Description
**Perceptions of the Pharmacy Profession**	*Expectations of Pharmacist’s Role (CP perception of patient and provider)*	Patients understand and respect the role of pharmacists and perceive pharmacists as very knowledgeable Resistance to increased provider collaboration exists as pharmacist continue to prove value
	*Need for Marketing pharmacy care services*	Pharmacists must promote their patient care services to raise awareness of their profession’s ability
	*Advocating for the Pharmacy Profession*	Pharmacists need to have expanded legislation to see change
	*Prioritizing Patient Care*	Developing personalized relationships with patients
**Reimbursement Models**	*Unsustainable Current Reimbursement Model*	Not adequately reimbursed for the provision of PCS
	*Current Progressive Models*	Currently receiving grant money for providing PCS Contracting with various organizations for reimbursement through providing innovative PCS
	*Future Progressive Models*	Increase contracting opportunities with CPESN to increase PCS reimbursement
**Provision of Patient Care Services**	*Barriers*	Inadequate resources to devote to PCS
	*Operational Concerns*	Balancing resources to devote to PCS
	*Opportunities*	Example: Educate patients during transitions of care such as hospital discharge
	*Quantifiable Outcomes*	Increased patient adherence score (e.g., – Proportion of Days Covered)
	*Perceived value of Patient Care Services*	Value: increased medication adherence (e.g., –adherence packaging, MTM)
**Social Determinants of Health**	*Personalized Approach*	Knowing the patient beyond their medication profile
	*Patient Barriers to Care*	Low health literacy (e.g., stockpiling medications, late or early refills Low Socioeconomic status (e.g., insurance coverage, affordability of medications) Access to adequate healthcare (e.g., no primary care provider, lack a transportation)
	*Pharmacy PCS Solutions*	Delivery—Helps patients who are home bound or do not have transportation Patient Education—Monitoring adherence and efficacy through follow-up phone calls

Abb. CP, Community Pharmacist; CPESN, Community Pharmacy Enhanced Service Network; MTM, Medication therapy management; PCS, Patient Care Services.

## Supplementary Materials

The following are available online at https://www.mdpi.com/2226-4787/8/3/172/s1.

## Appendix A

**Table A1:** Semi-structured interview guide with questions.

Domain	Questions
Domain 1: To collect the current level of commitment among community pharmacies to deliver patient care services.	What do you think is the current perception of a pharmacist in the healthcare system? How do you think pharmacists are integrated into an inter-professional collaborative team? In terms of the public’s point of view? In terms of other healthcare providers’ point of view? What services are you providing in addition to dispensing, and how are you being reimbursed for said services? Which of the patient care services offered at your pharmacy do you think make the largest impact in terms of improving patient outcomes? Discuss the top 3. What does your patient population value the most when it comes to the provision of patient care? Please state specific qualitative examples of improved patient health (ex. Hemoglobin A1C, Proportions of Days Covered (PDC) scores, etc.).
Domain 2: To assess the readiness of community pharmacies to deliver patient care services.	3. How did you set up your first patient care service at the pharmacy? escribe the process. What barriers did you encounter? 4. What barriers are preventing you from providing patient care services? Discuss the top 3. How have you addressed these barriers? Please state potential solutions to these barriers that you have not tried yet. 5. What are some additional resources that would benefit you and your pharmacy in helping provide patient care services?
Domain 3: To assess how community pharmacies address social determinants of health and address health disparities in underserved populations.	6. How does your pharmacy identify patients that are considered an underserved population? 7. What barriers are preventing you from providing adequate care to underserved populations at your pharmacy? 8. Of the patient care services provided at your pharmacy, which services are utilized the most by the underserved populations? Discuss the top 3. 9. What services would you like to incorporate into your pharmacy that would allow you to better serve underserved populations? 10. Please give an example of how your pharmacy addresses each part of the quadruple aim: Improving patient experience of care Improving the health of the population Reducing per capita cost of healthcare Reducing provider burnout/improving provider’s quality of life (Pharmacists)

## Appendix B

**Table A2:** Consolidated criteria for reporting qualitative studies (COREQ): 32-item checklist.

No. of Item	Guide Questions	Description
Domain 1: Research team and reflexivity		
Personal Characteristics		
1. Interviewer/facilitator	Which author/s conducted the interview or focus group?	Authors BQ and AM conducted all interviews.
2. Credentials	What were the researcher’s credentials? (e.g., PhD, MD)	Authors CD and DJ are pharmacy practice faculty; CD holds both a Doctor of Pharmacy and Master of Business Administration degrees; DJ holds both a Doctor of Pharmacy and Doctor of Philosophy in epidemiology; BQ and AM are both Doctor of Pharmacy Candidates; All are affiliated with the University at Buffalo School of Pharmacy and Pharmaceutical Sciences
3. Occupation	What was their occupation at the time of the study?	CD and DJ are pharmacists working as faculty; BQ and AM are students in a pharmacy program
4. Gender	Was the researcher male or female?	CD, DJ, and BQ are male; AM is female
5. Experience and training	What experience or training did the researcher have?	Investigators CD and DJ are both residency trained having received Doctor of Pharmacy Degrees from the University at Buffalo School of Pharmacy and Pharmaceutical Sciences; CD specialized in outpatient pharmacy innovation at the UNC Eshelman School of Pharmacy with quantitative and qualitative research experience; DJ received a PhD in epidemiology from the University at Buffalo School of Public Health and Health Professions and received in-depth research experience in both quantitative and qualitative methods; BQ and AM were both academic research assistants from clinical backgrounds and received formal training from investigators CD and DJ.
Relationship with participants		
6. Relationship established	Was a relationship established prior to study commencement?	Those whom participated in the interviews had previously participated in the 35-item cross sectional electronic survey, showing prior relationship to this study. A total of 48 potential interviewees self-identified based on their willingness to provide a follow-up interview from the initial survey. CD serves in a Board of Manager role for CPESN NY, LLC and CPESN NY, IPA, the same group as the source of participants. To manage the conflict, CD was removed from the interview, data collection, and part of the data analysis steps. Other members of the team acted ethically as to not disclose those involved. CD made no contact with study participants. The members of the research team involved in telephonic contact with study participants (BQ and AM) did not have any prior contact or interactions with study participants. The two interviewers (BQ and AM) were both academic research assistants from clinical backgrounds. Past professional background, experiences and prior assumptions were mitigated by this split team approach. Other members of the study team (CD and DJ) were removed from this process due to affiliations and only analyzed de-identified data. No contact between the principal investigators (CD and DJ) were made with the study participants.
7. Participant knowledge of the interviewer	What did the participants know about the researcher? (e.g., personal goals, reasons for doing the research)	The participants received an IRB-approved consent form with information about the study. It outlined the research team was from the University at Buffalo, goals of the research, methods of data collection, how information will be stored and used, and participant rights. Participants had this prior knowledge about the basis of the study due to completing the cross-sectional survey prior to interviews.
8. Interviewer characteristics	What characteristics were reported about the interviewer/facilitator? (e.g., bias, assumptions, reasons and interests in the research topic)	Due to participants being employed by CPESN affiliate entities, no contact between the principal investigators (CD and DJ) were made with the study participants. Interviewers (BQ and AM) were trained in qualitative research methodology prior to conducting the interviews. BQ and AM completed sufficient research about the interview topics to conduct the interviews.
Domain 2: Study Design		
9. Methodological orientation and theory	What methodological orientation was stated to underpin the study? (e.g., grounded theory, discourse analysis, ethnography, phenomenology, content analysis)	A qualitative study to conduct a semi-structured interview with open ended questions to elicit in-depth responses. A semi-structure interview guide was developed by the research team after seeking team expert input and completing a literature search. This can be found in Appendix A. The consolidated criteria for reporting qualitative studies (COREQ) guidelines were used to report qualitative research.
10. Sampling	How were participants selected? (e.g., purposive, convenience, consecutive, snowball)	A total of 48 potential interviewees self-identified based on their willingness to provide a follow-up interview from the initial survey. Participants were chosen based on geographical location to provide a diverse pool of interviewees.
11. Method of approach	How were participants approached? (e.g., face-to-face, telephone, mail, email)	Potential interviewees were recruited to participate via telephone by members of the research team (BQ and AM).
12. Sample size	How many participants were in the study?	Representation consisted of all CPESN NY chapters; Upstate New York (6), Western New York (4), and New York City (2). This was a total of 12 participants. The authors aimed to recruit participants from the three areas and stopped recruitment after consistent findings and perceptions were reached.
13. Non-participation	How many people refused to participate or dropped out? Reasons?	No participants refused or dropped out of the study.
14. Setting of data collection	Where was the data collected? (e.g., home, clinic, workplace)	Interviews were conducted via phone from the University at Buffalo in a closed private room.
15. Presence of non-participants	Was anyone else present besides the participants and researchers?	The phone interviews were conducted one-on-one by either BQ or AM and the recruited participant. No other individuals were present in the room or on the phone. Each researcher conducted six interviews.
16. Description of sample	What are the important characteristics of the sample? (e.g., demographic data, date)	Majority (11) of the study participants were pharmacy owners as shown in Table 1. Over half of the community pharmacies (*n* = 7) had a weekly prescription count of >1200. The average time devoted to PCS was 15 h/week and pharmacies on average spent 8 h/week addressing social barriers. Further details can be found in Section 3.1.
17. Interview guide	Were questions, prompts, guides provided by the authors? Was it pilot tested?	A semi-structure interview guide was developed by the research team after seeking expert input and completing a literature search. This can be found in Appendix A.
18. Repeat interviews	Were repeat interviews carried out? If yes, how many?	There were no repeat interviews conducted.
19. Audio/visual recording	Did the research use audio or visual recording to collect the data?	All interviews were digitally recorded and conducted in English. All files were stored on a password protected research computer according to the ethical standards of the University at Buffalo IRB.
20. Field notes	Were field notes made during and/or after the interview or focus group?	There was no note of field notes being taken as a result of this study.
21. Duration	What was the duration of the interviews or focus group?	The duration of the interviews with study participants ranged from 27 min to 99 min with an average length of 45 min.
22. Data saturation	Was data saturation discussed?	At a mid-point of the analysis of the qualitative data, a research meeting was conducted of all team members examining five de-identified transcripts and the codes created by the members of the team most closely involved in data collection and analysis (BQ and AM). As an independent check, the assignment of codes to the five de-identified transcripts was performed by other team members (CD and DJ). The result produced a codebook that would be used for further interviews. After seven additional interviews and analysis the research team concluded that data saturation was met due to consistent themes and findings.
23. Transcripts returned	Were transcripts returned to participants for comment and/or correction?	No transcripts were provided or returned to participants for comments.
Domain 3: Analysis and Findings		
24. Number of data coders	How many data coders coded the data?	Two of the authors (BQ and AM) read through the data files and independently coded the interview data. As an independent check, the assignment of codes to the five de-identified transcripts was performed by other team members (CD and DJ).
25. Description of the coding tree	Did authors provide a description of the coding tree?	Coding tree was facilitated by the use of a comprehensive chart forming the basis of the framework. Comparing data between the initial five participants allowed for the exploration of contextual meaning, while comparing across the data set facilitated the identification of key themes.
26. Derivation of themes	Were themes identified in advance or derived from the data?	The initial thematic analysis was conducted by the research team using the mid-point interview data to generate a set of codes that were based on the interview guide. After an additional seven interviews were conducted, transcribed, and coded, the research team met to discuss consensus themes.
27. Software	What software, if applicable, was used to manage the data?	Analysis and coding of the transcripts were supported by use of Microsoft Office Excel^®^ version 2019.
28. Participant checking	Did participants provide feedback on the findings?	After the transcripts were coded, a summary of findings was sent to three of the interview participants to provide feedback of relevance and contextual accuracy. The aim of this process was to make sure the interpretation of the findings was consistent with current experiences. The interview participants agreed and did not provide any changes to the findings.
29. Quotations presented	Were participant quotations presented to illustrate the themes/findings? Was each quotation identified? (e.g., participant number)	Themes are illustrated by participant quotations. Examples of quotes were used and identified as participant number such as, “Pharmacist 6.”
30. Data and findings consistent	Was there consistency between the data presented and the findings?	Previous community pharmacy qualitative work describing community pharmacy practice transformation, patient care services, provider collaboration, and alternative payment model shows the current dynamic model evolution.
31. Clarity of major themes:	Were major themes clearly presented in the findings?	After thorough analysis of the data, four themes identified by the research team include: (1) perceptions of pharmacy profession, (2) reimbursement models, (3) provision of patient care services, (4) social determinants of health. This is presented in Table 2.
32. Clarity of minor themes	Is there a description of diverse cases or discussion of minor themes?	Minor or subthemes are described in the results section, Table 2.

Abb. CPESN, Community Pharmacy Enhanced Service Network. Developed from: Tong, A.; Sainsbury, P.; Craig, J. Consolidated criteria for reporting qualitative research (COREQ): a 32-item checklist for interviews and focus groups [20].

## Appendix C

**Table A3:** Supplemental quotes.

Themes *(Subthemes)*	Quotes
**Perceptions of the Pharmacy Profession**	
*Expectations of Pharmacist’s Role (CP perception of patient and provider)*	*“…I believe we can play a critical role in lowering the overall healthcare costs of patients, but it must be recognized by other healthcare providers to get buy-in and it must be known to the public so that they can take advantage of it. We can do it one at a time when the patient comes up to the counter or when we look for opportunities, but if the patient doesn’t know then they won’t know to ask for it.” [Pharmacist 11]* *“…the public perception of pharmacy. You have major players in the retail field pushing for the “mcdonaldization” of pharmacy, so that is the kind of service people expect. In order to convince patients to sit down and take the time to meet with pharmacists, it would definitely be helpful if other members of the healthcare team referred patients to us, like doctors and nurses. I’ve spoken to other providers about this and they think it’s an okay idea but they don’t see so much value that they push their patients to do that.” [Pharmacist 3]*
*Need for Marketing pharmacy care services*	*“First thing is to find a person who can relay our story to patient, payer, and PBM, even though PBM is not listening. Historically, pharmacists are not comfortable about all their accomplishments and what they can do. We do things to help people and do not tell the whole story. If we do not tell people about what we do, it does not matter. If I had unlimited resources, I would hire a consultant to relay the pharmacies’ story to everyone – ACOs, law makers. I want someone to market their services to help move the needle forward**”** [Pharmacist 3]* * “The public is undereducated on the pharmacist’s role, what they can provide.” [Pharmacist 7]*
*Advocating for the Pharmacy Profession*	*“Getting involved in the profession and advocacy also leads to changes in legislation which can improve our practice of pharmacy.” [Pharmacist 10]* *“While healthcare dollars and spending continues to increase, it’s not the pharmacy making the money but the PBMs that are profiting. Until pharmacy is able to get provider status and be given a little bit more liberty legislatively to show what we can do with POC testing and other things, there really isn’t a desire for product reimbursement.” [Pharmacist 6]*
*Prioritizing Patient Care*	*“I think the personal service, taking time out and speaking to them [patients], answering all their questions and having them get a good grasp of what is going on with their medication regimen. Again the educational component is probably the thing they value the most. Also being able to know them on a personal level, it makes them feel comfortable with us. I think that goes a long way as opposed to the far other extreme of something like a mail order pharmacy where they [patients] don’t know any of the pharmacists, they probably get a different person every time they call—if they can even get through to a pharmacist in a timely fashion. I think just being available to them, being a familiar face, that type of this is what they [patients] would value the most.” [Pharmacist 8]* *“…[allows] us to provide these services and provide that quality of care that I strive for. Improving patient care has always been a priority.” [Pharmacist 1]*
**Reimbursement Models**	
*Unsustainable Current Reimbursement Model*	*“...it costs money to do these programs. We’re trying to do that to maximize our clinical effectiveness and hopefully get reimbursed for our services, not through PBMs but through other avenues. It might be a last-ditch effort to do as much as we can to make us more valuable.” [Pharmacist 4]* *“Reimbursement, there’s only so much you can do for free. Pharmacists aren’t cheap, and our time is very scarce. So, it’s disappointing when they don’t get paid for their time.” [Pharmacist 7]*
*Current Progressive Models*	*“I’ve heard of pharmacies receiving grants, which would be a huge benefit. We were in a blood pressure one which provided a fair amount of money for pharmacist’s assistance. Any government-funded programs or initiative where there is proper reimbursement for a pharmacist’s time would be some resources that we would be interested in.” [Pharmacist 8]* *“Even though you can’t classify our business as nonprofit, our goal is to help patients in our neighborhood and I should explore this avenue to see if organizations are willing to help pharmacies like mine through grant money to improve patient outcomes and services.” [Pharmacist 1]*
*Future Progressive Models*	*“Another thing is to get more contracts through CPESN and push those enhanced services and build a medical side of the healthcare pie for the services that pharmacists provide.” [Pharmacist 1]* *“Resources such as CPESN have been fantastic. I’ve learned so much about enhanced services and engaging payers and understanding how payers look at things such as services and how they pay for things such as extra services. Since CPESN, we’ve had a great education into the other side of what we do as pharmacists - the payment world that the PBMs and insurance folks are in.” [Pharmacist 2]*
**Provision of Patient Care Services**	
*Barriers*	*“Definitely cost. Reimbursements keep on bringing down our abilities to bring on new services.” [Pharmacist 2]* *“I’m trying to get more involved through organizations and programs like CPESN, prescribe wellness. They have platforms for integrating data through our pharmacy software systems to help increase star ratings. The problem is that they have tools to help, but everything’s an additional cost.” [Pharmacist 4]*
*Operational Concerns*	*“Probably number one would be staffing and time. That would probably be the biggest issue. A lot of these things take time, and time is money when it comes to staffing.” [Pharmacist 8]* *“Home delivery you encounter barriers every day because if we don’t establish policies around if a patient doesn’t answer the phone and we don’t have a safe place to leave medication, like we may have 30 or 40 deliveries per day that go to tenant buildings. So patients need to be home and that’s a challenge. Delivery business is always a challenge because you want to make sure you get the patient what they need when they need it but if they make it difficult for you, that’s a barrier. You can’t afford to send a driver to a place more than once in a day because it’s just not economically feasible. [PBMs are] not paying for delivery.” [Pharmacist 11]*
*Opportunities*	*“Having access to EMRs. We have one office that does that with us. It’s limited so that we can access labs in their EMR. I think partnering with technology companies to allow us to see some of that information and having provider’s offices see the value in that. It would cut down on some of the phone calls and questions we ask the office. It would allow us to run more thorough MTMs, CMR, identify gap therapy. There would be better communication between the primary care provider and the pharmacy, we both would benefit.” [Pharmacist 6]* *“We are perfectly positioned to be that go between in transitions of care, which is becoming a standard of care from a CMS standpoint. There is a significant revenue stream for primary care if they see patient within 7 days of discharge and 55% of hospital readmission are drug related which puts pharmacists in a perfect position to help with this role and hopefully get reimbursed.” [Pharmacist 10]*
*Quantifiable Outcomes*	*“The third service would be our asthma therapy and helping patients with this. This is a huge financial opportunity especially because we created a partnership with The American Lung Association. We provide peak flow and spacers for asthma patients so we can monitor their results and ensure they are using their inhalers properly. This can also help predict their exacerbations and help keep our patients healthier and track statistics to see how we are improving patient health.” [Pharmacist 10]* *“When we put patients on med sync and furthermore the adherence packaging, their PDC almost immediately goes to 100% with some outliers like discontinuation of meds, increase or decrease in dose, and it gets hard to compute the PDC at that point because you get some blurred lines around therapies like changes in therapies from one statin to another or one oral hypoglycemic to another and things such as that.” [Pharmacist 11]*
*Perceived value of Patient Care Services*	*“We had a patient on blister packaging who went down from mid-8 to mid-6 HgA1c score. The patient was on a ton of medications and was taking the medications all wrong prior to the blister packaging. With counseling and education on how to use the blister packaging, the patient was able to see the 2-point drop in HgA1c.” [Pharmacist 5]* *“Then with medication synchronization, some barriers are that you have patients that understand it or say they understand it then you implement it and before you know it 4 months goes by and you find out that they’re stock piling medication because they’re not taking it properly. Months into this they realize they have all this extra medication and they tell us to stop sending it to them. That was a barrier that pushed that patient into medication adherence packaging because we realized that even though we were filling the medication and PDC scores were going up, the patient wasn’t necessarily adherent or compliant to their medication schedule. That was a barrier at first, and pushes folks into the adherence packaging which forces them to become more compliant with their schedules.” [Pharmacist 11]*
**Social Determinants of Health**	
*Personalized Approach*	*“The fact that we really incorporate them into their own care, with our med sync program they get multiple phone calls from pharmacy. I think they really feel that we are really looking out for them as far as their overall health and wellbeing. They get at least 3 phone calls a month from us, a consultation with the pharmacist to reinforce how important each medication is. They just really feel empowered themselves.” [Pharmacist 6]* *“Some patients are proud and don’t want help or anything that might be perceived as charity. You need to develop a relationship with a patient before you ask them if they want to try a program. I assume that my patient is going to need to utilize the services that I offer, but if I think they are that type of person I dial it back.” [Pharmacist 1]*
*Patient Barriers to Care*	*“We offer charge accounts for patients living check to check can pick up their prescriptions at any time during the month then pay at the end of the month. This doesn’t seem like an enhanced service because we have been doing it for so long, but it is.” [Pharmacist 1]* *“We tried pushing for compliance packaging but a big barrier to healthcare among underserved populations is low health literacy. A lot of patients don’t understand that they need to be constantly treating their chronic disease states, they can’t just take a few things and be done with it. It’s just really difficult to shake those misconceptions. It needs a coordinated effort from all members of the healthcare team.” [Pharmacist 2]*
*Pharmacy PCS Solutions*	*“The compliance (adherence) packaging definitely helps many of my patients. I had a patient who went from completely non-compliant and unreliable to compliant enough where their liver specialists decided to put them on treatment for Hep C and now they’re cured of Hep C.” [Pharmacist 2]* *“Our diet program has done more than anything. We had a patient who was a type 2 diabetic on an insulin pump. She’s been on our wellness program for about 4 months and they just turned off her insulin. She’s needs no insulin, not on any diabetic meds right now, and is just controlling it with diet. We had patients with sugars around 300 that we got controlled. Our goal with the wellness program is not so much the weight loss but to cut back on patient’s meds.” [Pharmacist 5]*

Abb. ACO, Accountable Care Organization; CP, Community Pharmacist; CMR, Comprehensive medication review; CMS, Centers for Medicaid and Medicare services; CPESN, Community Pharmacy Enhanced Service Network; EMR, Electronic Medical Record; Hep C, Hepatitis-C; HgA1c, hemoglobin A1c; Med sync, Medication Synchronization; MTM, Medication therapy management; PBM, Pharmacy Benefit Manager; PDC, Proportion of Days Covered; PCS, Patient Care Services.
