# Supplementary material for: Community Pharmacists’ Perceptions of Patient Care Services within an Enhanced Service Network
Source: Pharmacy (Basel). 2020 Sep 16;8(3):172. doi: 10.3390/pharmacy8030172 (PMC7559089; doi:10.3390/pharmacy8030172)
Supplement: Supplementary file 1 [file pharmacy-08-00172-s001.pdf]

Title: Community Pharmacists’ Perceptions of Patient Care Services within an Enhanced Service Network

Survey Host by Google® Docs

1. **Consent Statement (required question) (IRB statement provided, removed for posting)**
   Mark only one oval.

   ◯ I accept

2. **Please state the name of your pharmacy below:**

3. **What is your role at the pharmacy? (Select all that apply)**
   Check all that apply.

   ☐ Owner
   ☐ Pharmacist
   ☐ Supervising Pharmacist
   ☐ Manager
   ☐ Other: ______________________

4. **What is the average weekly prescription volume?**
   Mark only one oval.

   ◯ 0-200
   ◯ 201-400
   ◯ 401-600
   ◯ 601-800
   ◯ 801-1,000
   ◯ 1,001-1,200
   ◯ >1,200

5. **Do you currently partner with a provider collaboration to deliver clinical services? (ex: consultative services with primary care physician) (Select only one option)**
   Mark only one oval.

   ◯ No, no providers are currently accessible.
   ◯ No, currently looking for provider partners.
   ◯ Yes, currently participate in provider partnership.
   ◯ Unaware of partnerships with providers to deliver clinical services

6. **How much time per week does your pharmacy currently devote to patient care services?**

Mark only one oval.

- 1-5 hours
- 6-10 hours
- 11-15 hours
- 16-20 hours
- 21-25 hours
- 25+ hours

7. **What is the minimal amount of time per week that your pharmacy is willing to devote to patient care services?**

Mark only one oval.

- 1-5 hours
- 6-10 hours
- 11-15 hours
- 16-20 hours
- 21-25 hours
- 25+ hours

8. **Are you willing and/or able to collaborate with other members of the health care team including physicians and other prescribers, care managers, nurses and social workers—all with the goal of improving outcomes? (Select all that apply)**

Check all that apply.

- [ ] I am willing to collaborate with other members of the health care team
- [ ] I am able to collaborate with other members of the health care team
- [ ] I am not willing nor able to collaborate with other members of the health care team

9. **Do you currently partner with a third party/other entity that is incentivizing you to deliver clinical services? (ex: adherence interventions, targeted medication interventions) (Select only one option)**

Mark only one oval.

- No, no insurance payers are accessible.
- No, currently looking for insurance payer partners.
- Yes, currently participate in insurance payer partnership.
- Unaware of partnerships with insurance payers to deliver clinical services

10. **Please select the patient care services that are currently offered by your pharmacy? (Select all that apply)**

Check all that apply.

- [ ] 24-hour emergency services
- [ ] Adherence Packaging
- [ ] Collection of vital signs
- [ ] Disease state management program
- [ ] Durable Medical Equipment
- [ ] Home delivery
- [ ] Home mail option (ex: Fedex delivery)
- [ ] Home Visits
- [ ] Immunizations
- [ ] Referring immunizations that cannot be given at the pharmacy
- [ ] Medication therapy management/comprehensive medication review
- [ ] Medication Synchronization
- [ ] Medication waste management program
- [ ] Multi-lingual capability
- [ ] Naloxone dispensing
- [ ] Non-sterile compounding
- [ ] Nutritional counseling (ex: Diabetes diet, DASH diet)
- [ ] Adherence Coaching
- [ ] Point of care testing
- [ ] Smoking cessation
- [ ] Standardized Assessments (PHQ-9)
- [ ] Specialty Medication (ex: REMs Program Certified Clozapine)
- [ ] Medication Reconciliation
- [ ] Other: ______________________

11. **Please select the patient care services that you are currently not conducting but are willing to provide with proper incentives? (Select all that apply)**

Check all that apply.

- [ ] 24-hour emergency services
- [ ] Adherence Packaging
- [ ] Collection of vital signs
- [ ] Disease state management program
- [ ] Durable Medical Equipment
- [ ] Home delivery
- [ ] Home mail option (ex: Fedex delivery)
- [ ] Home Visits
- [ ] Immunizations
- [ ] Referring immunizations that can not be given at the pharmacy
- [ ] Medication therapy management/comprehensive medication review
- [ ] Medication Synchronization
- [ ] Medication waste management program
- [ ] Multi-lingual capability
- [ ] Naloxone dispensing
- [ ] Non-sterile compounding
- [ ] Nutritional counseling (ex: Diabetes diet, DASH diet)
- [ ] Adherence Coaching
- [ ] Point of care testing
- [ ] Smoking cessation
- [ ] Standardized Assessments (PHQ-9)
- [ ] Specialty Medication (ex: REMs Program Certified Clozapine)
- [ ] Medication Reconciliation
- [ ] Other: ______________________________

12. **Please state how much you agree with each statement:**

Mark only one oval per row.

	Strongly Agree	Agree	Neutral	Disagree	Strongly Disagree
Patients can contact the pharmacy through multiple methods of communication. (ex: in person, phone, email, etc.)	⬭	⬭	⬭	⬭	⬭
Patients are contacted through mostly text message and/or email.	⬭	⬭	⬭	⬭	⬭
Patients are contacted through mostly phone calls and/or in-person conversation.	⬭	⬭	⬭	⬭	⬭
Patients are kept up to date on changes in pharmacy services.	⬭	⬭	⬭	⬭	⬭
Patients are targeted for participation in clinical programs.	⬭	⬭	⬭	⬭	⬭
Patients are included in clinical programs with the ability to opt-out.	⬭	⬭	⬭	⬭	⬭

13. **My pharmacy is actively involved in these professional pharmacy organizations: (Select all that apply)**

Check all that apply.

- ☐ PSSNY
- ☐ Local PSSNY Affiliate (PAWNY, PSR, CAPS)
- ☐ APhA
- ☐ NCPA
- ☐ My pharmacy is not involved in any organizations
- ☐ Other: ______________________

14. **I would like to learn more about the following professional pharmacy organizations: (Select all that apply)**

Check all that apply.

- ☐ PSSNY
- ☐ Local PSSNY Affiliate (PAWNY, PSR, CAPS)
- ☐ APhA
- ☐ NCPA
- ☐ Other: ______________________

15. **Is your pharmacy affiliated with a Community Pharmacy Enhanced Services Network? (Select all that apply)**

Check all that apply.

- [ ] CPESN of WNY
- [ ] CPESN of Upstate
- [ ] CPESN of NYC
- [ ] Yes, there is a network but my pharmacy is not a member.
- [ ] I don't know enough about patient care services to be involved
- [ ] No network exists in the area at this time.

16. **I would like to learn more about the following Community Pharmacy Enhanced Services Network? (Select all that apply)**

Check all that apply.

- [ ] CPESN of WNY
- [ ] CPESN of Upstate
- [ ] CPESN of NYC
- [ ] Yes, there is a network but my pharmacy is not a member.
- [ ] I don't know enough about patient care services to be involved
- [ ] No network exists in the area at this time.

17. **Based on proper incentives, which of the following apply to patient care services for your pharmacy? (Select all that apply):**

Check all that apply.

- [ ] I would conduct if there was no reimbursement/incentive to do so
- [ ] I would conduct if there was reimbursement/incentive that covered the costs (time) of the intervention (breakeven)
- [ ] I would conduct if there was reimbursement/incentive that allowed for profit during the intervention
- [ ] My pharmacy is already conducting patient care services
- [ ] I don't know enough about patient care services to be involved

18. **If proper reimbursement is available, please state how much you agree with each statement about hiring additional staff to provide patient care services:**

Mark only one oval per row.

	Strongly Agree	Agree	Neutral	Disagree	Strongly Disagree
I would be willing to hire additional pharmacy interns	◯	◯	◯	◯	◯
I would be willing to hire additional technicians	◯	◯	◯	◯	◯
I would be willing to hire an additional pharmacist	◯	◯	◯	◯	◯
I am not willing to hire additional staff due to financial burden	◯	◯	◯	◯	◯
I am not willing to hire additional staff because we have adequate staff to devote to services	◯	◯	◯	◯	◯

19. **If proper incentives were in place, how much additional time are you willing to devote to these patient care services per week? (select only one option)**

Mark only one oval.

- ◯ I do not have additional time to devote to these services
- ◯ 1-5 hours
- ◯ 6-10 hours
- ◯ 11-15 hours
- ◯ 16-20 hours
- ◯ 21-25 hours
- ◯ 25+ hours

20. **Does the pharmacy computer software have data collection and analysis capabilities? (ex: ability to recommend patients for clinical program based on prescription data) (Select only one option)**

Mark only one oval.

- ◯ Yes, the software has the capabilities and are utilized to improve clinical programs and pharmacy operations.
- ◯ Yes, the software has the capabilities but the pharmacy is not using it to the fullest extent.
- ◯ No, the software does not currently offer data collection and analysis functions.
- ◯ Other: ______________________

21. **How are patients targeted for enrollment in clinical programs? (Select all that apply)**
Check all that apply.

- [ ] Staff recommendation
- [ ] Patient self-referral
- [ ] Opt-out programming (e.g. all patients included unless choose to leave program)
- [ ] Data mining/Computer data analysis recommendation
- [ ] Insurance payer recommendation
- [ ] Physician recommendation
- [ ] Other: ______________________

22. **The pharmacy engages prescribers in collaborative care through: (Select all that apply)**
Check all that apply.

- [ ] Scheduled meetings (in-person, teleconference)
- [ ] Co-meetings with Patient
- [ ] Standardized communication documents (ex: MTM recommendations form)
- [ ] Routine updates of patient progress
- [ ] Other: ______________________

23. **Select the adherence tools used by the pharmacy: (Select all that apply)**
Check all that apply.

- [ ] Text message alerts and reminders
- [ ] Email alerts and reminders
- [ ] Automated phone call alerts and reminders
- [ ] Automated/scheduled filling
- [ ] Pill boxes
- [ ] Medication synchronization programs
- [ ] Commingle packaging
- [ ] Other: ______________________

24. **Is there a care coordination program for disease state management? (A care coordination program organizes the needed healthcare services in collaboration with other healthcare entities including physicians and insurance payers.) (Select only one option)**
Mark only one oval.

- ( ) Yes, the pharmacy has a structured care coordination program.
- ( ) A care coordination program is being constructed currently.
- ( ) No, the pharmacy does not have a structured care coordination program.
- ( ) Other: ______________________

25. **Are patients engaged in their care through scheduled appointments at the pharmacy? (Select only one option)**

Mark only one oval.

- ◯ Yes, the pharmacy currently uses an appointment-based model for clinical programs.
- ◯ Yes, the pharmacy is initiating an appointment-based model for clinical programs.
- ◯ No, the pharmacy does not utilize an appointment-based model for clinical programs
- ◯ Other: ____________________

26. **Which social barriers exist with patients at your pharmacy that prevent optimal care? (Select all that apply)**

Check all that apply.

- ☐ Transportation
- ☐ Low income
- ☐ High medication costs
- ☐ Low education level
- ☐ Exposure to crime or violence
- ☐ Language barrier
- ☐ Lack of insurance coverage
- ☐ Culture/religious differences
- ☐ Lack of primary care physician
- ☐ Other: ____________________

27. **What percentage of your patients experience the social barriers listed above?**

Mark only one oval.

- ◯ 0-10%
- ◯ 11-20%
- ◯ 21-30%
- ◯ 31-40%
- ◯ 41-50%
- ◯ 51-60%
- ◯ 61-70%
- ◯ 71-80%
- ◯ 81-90%
- ◯ 91-100%

28. **Currently how many hours per week are devoted to addressing the social barriers experienced at your pharmacy?**

Mark only one oval.

- 1-5
- 6-10
- 11-15
- 16-20
- 20+

29. **Please state how much you agree with each statement**

Mark only one oval per row.

	Strongly Agree	Agree	Neutral	Disagree	Strongly Disagree
Our pharmacy asks our patients if there are reasons why they are not adherent to their medication.					
Our pharmacy addresses the social barriers experienced at our pharmacy.					
Our pharmacy is adequately staffed to help patients with the social barriers experienced at our pharmacy.					
Our pharmacy is willing to alter workflow to help address social barriers experienced at our pharmacy.					
Our pharmacy is willing to hire additional staff to address the social barriers experienced at our pharmacy.					
Our pharmacy is providing adequate care for hthe underserved population at our pharmacy.					

30. **What resources do you use to help with social factors affecting patient health?**
Check all that apply.

- [ ] Home delivery
- [ ] Prescription manufacturer coupons
- [ ] Offer counseling on medication education
- [ ] Educational Pamphlets
- [ ] Medication therapy management
- [ ] Translation phones
- [ ] Recommending more cost-effective medications to patients and providers
- [ ] Referral to other healthcare providers (ex: nurse, social worker)
- [ ] Referral to other organizations (ex: mental health facility)
- [ ] Other: ______________________________

31. **My pharmacy pursues the quadruple aim by (select all that apply):**
Check all that apply.

- [ ] Improving patient experience of care
- [ ] Improving the health of the population
- [ ] Reducing per capita cost of health care
- [ ] Reducing provider burnout/improving provider's quality of life

32. **An individual from our research team will be conducting follow up interviews to assess the motivation of pharmacies in conducting patient care services. We are also looking to assess what pharmacies are doing to care for their underserved population. Would you be willing to participate in a follow-up interview?**
Mark only one oval.

- ( ) Yes
- ( ) No

If you answered yes to the previous question, please provide <u>your contact information below:</u>

33. **Name:**

34. **Email:**

35. **Phone Number:**
